# Using Mobile Electroencephalography and Actigraphy to Diagnose Attention-Deficit/Hyperactivity Disorder: Case-Control Comparison Study

**DOI:** 10.2196/12158

**Published:** 2020-06-19

**Authors:** Kuo-Chung Chu, Hsin-Ke Lu, Ming-Chun Huang, Shr-Jie Lin, Wen-I Liu, Yu-Shu Huang, Jen-Fu Hsu, Chih-Huan Wang

**Affiliations:** 1 Department of Information Management National Taipei University of Nursing and Health Sciences Taipei Taiwan; 2 Department of Information Technology Taipei City Government Taipei Taiwan; 3 Department of Electrical, Computer, and Systems Engineering Case Western Reserve University Cleveland, OH United States; 4 Department of Computer Center Cheng Hsin General Hospital Taipei Taiwan; 5 Department of Nursing National Taipei University of Nursing and Health Sciences Taipei Taiwan; 6 Department of Child Psychiatry and Sleep Center Chang Gung Memorial Hospital and Chang Gung University Taoyuan Taiwan; 7 Division of Neonatology Department of Pediatrics Chang Gung Memorial Hospital and Chang Gung University Taoyuan Taiwan; 8 Department of Psychology Zhejiang Normal University Zhejiang China

**Keywords:** actigraphy, ADHD, attention deficit disorder with hyperactivity, clinical decision-making, electroencephalography, neuropsychological tests

## Abstract

**Background:**

Children with attention-deficit/hyperactivity disorder (ADHD), a neurobehavioral disorder, display behaviors of inattention, hyperactivity, or impulsivity, which can affect their ability to learn and establish proper family and social relationships. Various tools are currently used by child and adolescent psychiatric clinics to diagnose, evaluate, and collect information and data. The tools allow professional physicians to assess if patients need further treatment, following a thorough and careful clinical diagnosis process.

**Objective:**

We aim to determine potential indicators extracted from a mobile electroencephalography (EEG) device (Mindset; NeuroSky) and an actigraph (MotionWatch 8; CamNtech) and to validate them for diagnosis of ADHD. The 3 indicators are (1) attention, measured by the EEG; (2) meditation, measured by the EEG; and (3) activity, measured by the actigraph.

**Methods:**

A total of 63 participants were recruited. The case group comprised 40 boys and 9 girls, while the control group comprised 5 boys and 9 girls. The groups were age matched. The test was divided into 3 stages—pretest, in-test, and posttest—with a testing duration of 20 minutes each. We used correlation analysis, repeated measures analysis of variance, and regression analysis to investigate which indicators can be used for ADHD diagnosis.

**Results:**

With the EEG indicators, the analysis results show a significant correlation of attention with both hit reaction time (RT) interstimulus interval (ISI) change (*r*=–0.368; *P*=.003) and hit standard error (SE) ISI change (*r*=–0.336; *P*=.007). This indicates that the higher the attention of the participants, the smaller both the hit RT change and the hit SE ISI change. With the actigraph indicator, confidence index (*r*=0.352; *P*=.005), omissions (*r*=0.322; *P*=.01), hit RT SE (*r*=0.393; *P*=.001), and variability (*r*=0.351; *P*=.005) were significant. This indicates that the higher the activity amounts, the higher the impulsive behavior of the participants and the more target omissions in the continuous performance test (CPT). The results show that the participants with ADHD present a significant difference in activity amounts (*P*<0.001). The actigraph outperforms the EEG in screening ADHD.

**Conclusions:**

When the participants with ADHD are stimulated under restricted conditions, they will present different amounts of activity than in unrestricted conditions due to participants’ inability to exercise control over their concentration. This finding could be a new electronic physiological biomarker of ADHD. An actigraph can be used to detect the amount of activity exhibited and to help physicians diagnose the disorder in order to develop more objective, rapid auxiliary diagnostic tools.

## Introduction

Children with attention-deficit/hyperactivity disorder (ADHD), a neurobehavioral disorder, display behaviors of inattention, hyperactivity, or impulsivity, which can affect their ability to learn and establish proper family and social relationships. Children with ADHD can develop depression or behavioral problems, such as oppositional defiant disorder and conduct disorder [1]. Furthermore, children with ADHD may develop comorbid conditions like Tourette syndrome, learning disabilities, emotional disorders, sleep disorders, and anxiety disorders. Such comorbidities can aggravate ADHD, and these children may become more difficult to discipline and may appear to defy adults, annoy others deliberately, and show ill temper and other poor behavior; they may even become more difficult to treat [2,3].

Currently, diagnostic assessments such as the Wechsler Intelligence Scale for Children, computer-operated continuous performance test (CPT), the Gordon Diagnostic System (GDS) (Gordon Systems Inc), and the Wisconsin Card Sorting Test (Psychological Assessment Resources Inc) are used by child and adolescent psychiatric outpatient departments in hospitals [4,5]. In these assessments, parents are asked to respond to the child’s behavior using a Swanson, Nolan and Pelham scale. The rating scale used by physicians to diagnose ADHD is mainly based on the Diagnostic and Statistical Manual of Mental Disorders, Fifth Edition (DSM-V) diagnostic criteria. Patients can only be correctly diagnosed through multiple information sources, including the physician’s clinical evaluation, supplementary computer tests, scales filled out by their parents and teachers, a psychologist’s assessment, and outpatient observation symptoms, among other tools.

Many years ago, the activity recorder became prominent in our daily lives, from the early development of the simple pedometer to the current more sophisticated exercise tracker and activity intensity recorder. Wearers use these for daily activity management and medical assessment to measure and understand their quantitative body energy consumption, record behavior, and assess sleep quality. Medical-grade recorders have been used by many researchers to record the activities of the wearer’s circadian rhythm, as well as to help physicians by providing comparative and analytical behavioral patterns to understand objective sleep quality [6-12].

Brainwaves, on the other hand, can be used as a medical tool for carrying out differential diagnosis and to help physicians diagnose or exclude several illnesses [13-15]. They help provide an objective and more rapid understanding of the physical and mental status of individual patients. The brain is composed of neurons that perform electrical conduction activities while we think, imagine, observe, and perform many other mental activities, which produce weak electromagnetic waves. To observe these brain waves, a noninvasive brain wave acquisition method is used that amplifies brain waves with the help of electrode chips.

Diagnosing ADHD is time-consuming and can be complicated, as parents often need to make an appointment and schedule a hospital visit. As the development of the brain wave and activity recorder has gradually matured, it has become necessary to look for relevant indicators in brain waves and activity amounts in children with ADHD. Despite the current CPT, Test of Variables of Attention, GDS, and other auxiliary diagnostic tools, more convenient assessment tools are still needed. Furthermore, as the tests are boring, the process cannot be conducted if a child is not willing to cooperate. Therefore, the purpose of this study is to develop a more objective and faster assessment tool with electroencephalography (EEG) and actigraphy.

## Methods

### Research Tools

This study aims to explore the relationships among brain waves, activity amounts, and CPT indicators through a correlation analysis. The CPT [16] was used as the assessment tool to obtain various indicators of ADHD diagnosis reports, a mobile EEG device (MindSet; NeuroSky) [17] was used to obtain attention and meditation values in brain waves, and an actigraph (MotionWatch 8; CamNtech) was used to obtain information about activity amounts in the test. There have been a lot of studies focusing on detecting the attention and meditation values from EEG [18-21]. Attention is defined as the state of focus on relevant aspects of the environment, while meditation is defined as the state of relaxation in both the body and the mind.

The CPT is a set of assessment tools that use the computer as a platform. Children aged 6 to 12 years were recruited as research participants. The actigraph [22] was worn on the nondominant hand to cope with the use of similar sleep recorders.

### Study Design

#### Participants

This study was conducted with the permission of the Institutional Review Board of Chang Gung Memorial Hospital (No. 104-5397B). We defined an α of .05, power of 0.8, and effect size of Cohen *d*=0.8 for correlation analysis. The sample size of 12 was determined using G*Power (version 3.1.9.4; Heinrich-Heine-Universität Düsseldorf).

The participants and their parents signed the written consent form. The study was conducted from September 24, 2015, to September 23, 2016. A total of 63 participants, 45 boys and 18 girls, completed the test. The case group consisted of 49 participants with ADHD diagnosed by professional physicians. Participants were divided into a case group or control group depending on diagnosis of ADHD, and the groups were age matched.

#### Diagnosis Determination and Acceptance Criteria

**Figure 1:**
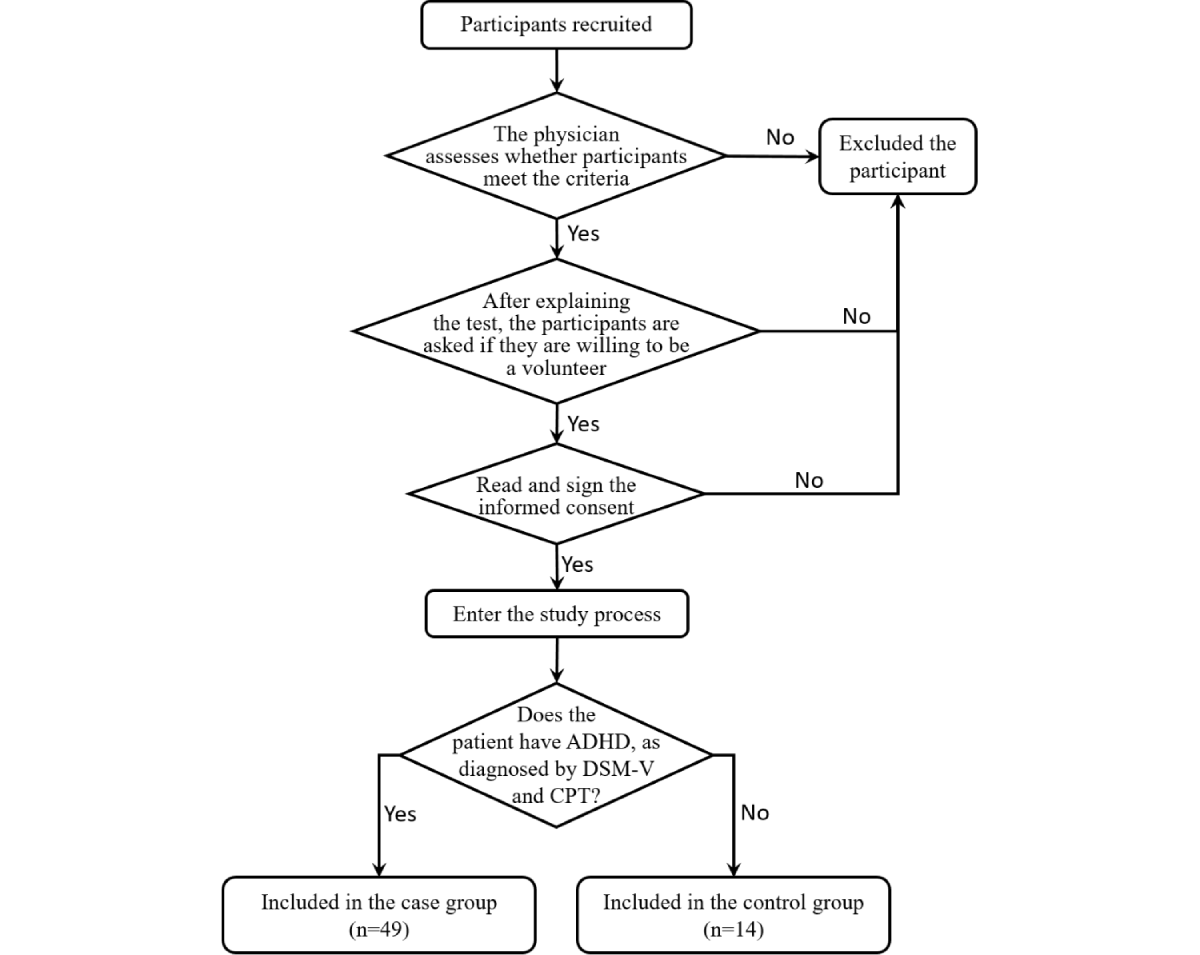
Flow chart of the participant inclusion and exclusion process. ADHD: attention-deficit/hyperactivity disorder. CPT: continuous performance test. DSM-V: Diagnostic and Statistical Manual of Mental Disorders, Fifth Edition.

In this study, participants were recruited from the hospital’s outpatient department and from elementary school. All participants were diagnosed and screened by professional pediatric psychiatrists, as shown in Figure 1.

The case group was made up of children with ADHD. The inclusion criteria were (1) participants who were willing to stop taking any psychotropic drugs or drugs that would affect the detection of cognitive functions 7 days before the test, (2) participants who could stabilize their emotions during the test, (3) participants who had been diagnosed with ADHD based on a CPT, and (4) participants who wanted to voluntarily participate in the test and had already signed a written consent form.

The control group was made up of children aged 6 to 12 years without ADHD who had normal growth development. The inclusion criteria were (1) participants who had not consumed any drugs that would affect the detection of cognitive functions 7 days prior to the test, (2) participants who could stabilize their emotions during the test, (3) participants who had taken a CPT and were found not to have ADHD, and (4) participants who wanted to voluntarily participate in the test and had already signed a written consent form.

The exclusion criteria for both the case group and the control group were (1) participants with low intelligence (a full intelligence quotient less than 70 points, as diagnosed by physicians); (2) participants with major brain diseases, severe psychiatric disorders, drug abuse, physical disabilities, or physical illnesses; (3) participants who had consumed drugs that would affect the cognitive functions measured by the test in the past 7 days; and (4) participants who did not sign the consent form.

#### Study Design and Evaluation

In this study, there were 63 participants, aged 6 to 12 years in 2 age-matched groups. Mean age, standard deviation, and a significance test are shown in Table 1.

The hardware used in the study was (1) a mobile EEG device, (2) a notebook computer, and (3) an actigraph. The software used in this study included (1) Windows 7 (Microsoft Corp), (2) ThinkGear Connector (NeuroSky), (3) Visual Studio 2010 (Microsoft Corp), (4) an actigraph resolution program (CamNtech), and (5) CPT II (Pearson Education).

**Table 1:** Descriptive statistics of the participants.

Characteristic		Case group (n=49)	Control group (n=14)	*P* value
Age, mean (SD)		8.69 (1.71)	8.18 (1.84)	.36
**Gender**				
	Male, n (%)	40 (82)	5 (36)	
	Female, n (%)	9 (18)	9 (64)	

The tests on the participants were administered by assistants, psychiatrists, and graduate students at National Taipei University of Nursing and Health Sciences. They received complete CPT standard training on the test procedures under the guidance of physicians, and all of them understood the test operating system and examiner guidelines. The examiner guidelines were (1) give clear guidance using clear language and ensure that participants can understand; (2) unless necessary, do not talk to the participants during the test; (3) if encountering a participant with severe hyperactive symptoms, use a persuasive manner to prevent them from standing up and leaving the test site; and (4) should any incident occur during the test process, the safety of the participants is the priority.

#### Research Process

The tests were conducted individually. Taking a total of 1 hour, the whole process was divided into 3 stages of 20 minutes each. Attention and meditation brain waves were recorded and activity levels were measured during all 3 stages. The test procedures for the case and control groups were the same. To reduce unnecessary interference, mobile phones, desktop computers, and other electronic equipment were turned off throughout the entire test. The study assessed brain waves and activity amounts in both the test with stimulation (stage 2) and the tests without stimulation (stage 1 and stage 3).

In stage 1 (pretest without stimulation), a parent brought the child to the test site and listened to a briefing about the test process. The participant was then helped to put on wearable devices to collect data from both the EEG and the actigraph, and the participant’s parent was asked to fill out the scale form to complete the pretest preparation. This was a free activity environment.

In stage 2 (in-test with stimulation), data were continuously collected while the participants were stimulated by the CPT. This was a test environment.

In stage 3 (posttest without stimulation), data were continuously collected after the CPT. The participants completed the test, the scale forms were collected from the parents, and the wearable devices were taken off of the participants. This was a free activity environment.

### Statistical Methods

Statistical analyses were performed using IBM SPSS 22.0. We conducted correlation analysis to compare the values collected from the EEG and the actigraph to the CPT parameters of impulsivity, alertness, etc. We conducted repeated measures of analysis of variance (ANOVA) for interaction effects and post hoc analysis for differences. To investigate the confounding effect, we applied regression analysis. The threshold of significance was preset to *P*=.05.

## Results

### Correlation Analysis

Correlation analysis was completed to assess the values extracted from the tools (the EEG’s brain waves and the actigraph’s activity amounts) and to validate the CPT indicators for diagnosis of ADHD in stage 2. Table 2 shows the correlation coefficient (*r*) between CPT indicators, EEG values, and actigraph values. With the EEG, correlation analysis only shows a significant correlation of attention with hit reaction time (RT) interstimulus interval (ISI) change (*r*=–0.368; *P*=.003) and hit standard error (SE) ISI change (*r*=–0.336; *P*=.007). This indicates that the higher the attention of the participants, the smaller both the hit RT change and the hit SE ISI change. With the actigraph, there is significant correlation of activity levels with 4 indicators: confidence index (CI) (*r*=0.353; *P*=.005), omissions (*r*=0.322; *P*=.01), hit RT standard error (*r*=0.393; *P*=.001), and variability (*r*=0.351; *P*=.005). This indicates that the higher the activity amounts, the higher the impulsive behavior of the participants and the more target omissions in the CPT. CI that fits clinical diagnosis is significant. As a result, the participants may possibly be screened for ADHD with an actigraph.

**Table 2:** Correlation of indicators between CPT, EEG, and actigraph in stage 2.

CPT^a^ indicator	Sensor						
	EEG attention		EEG meditation			Actigraph activity	
	*r*	*P* value	*r*	*P* value	*r*		*P* value
CI	–0.215	.09	0.053	.68	0.353		.005
Omissions	–0.012	.92	–0.054	.68	0.322		.01
Commissions	–0.045	.73	–0.086	.50	0.081		.53
Hit RT^b^	–0.230	.07	0.208	.10	0.169		.19
Hit RT SE	–0.244	.05	0.041	.75	0.393		.001
Variability	–0.216	.09	–0.106	.41	0.351		.005
Detectability (d')	–0.050	.70	–0.095	.46	0.137		.29
Response Style (β)	–0.044	.73	0.066	.61	0.126		.33
Perseverations	–0.125	.33	–0.105	.41	0.199		.12
Hit RT block change	–0.135	.29	0.073	.57	0.161		.21
Hit SE block change	–0.164	.20	–0.111	.39	0.180		.16
Hit RT ISI^c^ change	–0.368	.003	0.029	.82	0.241		.06
Hit SE ISI change	–0.336	.007	–0.046	.72	0.200		.12

^a^CPT: continuous performance test.

^b^RT: reaction time.

^c^ISI: interstimulus interval.

### Repeated Measures ANOVA

Based on the results of correlation analysis, the participants can be screened for ADHD by the activity indicator. In order to confirm the discrimination ability of the indicator, we conducted repeated measures ANOVA. Table 3 conforms to a spherical pattern in the within-subject test (*P*=.37). Table 4 shows significance with both within-subject (*P*<.001) and between-subject (*P*=.04) designs and no interaction effect between the group and the stage (*P*=.77).

We also conducted a post hoc test. Table 5 demonstrates significant difference between the 2 groups in the 3 stages. For the case group, there were significant differences among the three stages (stage 3>stage 1>stage 2), while only stage 2 differences were significant in the control group (stage 3=stage 1>stage 2). The analysis results show that the participants with ADHD can be screened because the difference in activity amounts is significant.

**Table 3:** Mauchly's test of sphericity in within-subject test^a^.

Group	Mean (SD)		
	Stage 1	Stage 2	Stage 3
Case (n=49)	6.717 (2.949)	1.795 (1.309)	7.562 (2.918)
Control (n=14)	5.530 (2.381)	0.911 (0.436)	6.057 (2.838)

^a^*P*=.37.

**Table 4:** Significance of the activity indicator in the 3 stages with respect to group.

Source		Sum of square	Mean square	*F* (*df*)	*P* value
**Subject**					
	Group	46.183	46.183	4.632 (1,61)	.04
	Residual	608.22	9.971	N/A^a^	N/A
**Stage**					
	Stage	770.011	385.005	97.644 (2,122)	<.001
	Group×stage	2.101	1.051	0.266 (2,122)	.77
	Residual	481.042	3.943	N/A	N/A
Sum		1907.557	N/A	N/A	N/A

^a^N/A: not applicable.

**Table 5:** Post hoc test after repeated measures ANOVA.

Group, Stage I, and Stage J			Mean difference	*P* value
**Case^a^**				
	**1**			
		2	4.922	<.001
		3	–0.845	.03
	**2**			
		1	–4.922	<.001
		3	–5.767	<.001
	**3**			
		1	0.845	.03
		2	5.767	<.001
**Control^b^**				
	**1**			
		2	4.627	<.001
		3	–0.519	.54
	**2**			
		1	–4.627	<.001
		3	–5.146	<.001
	**3**			
		1	0.519	.54
		2	5.146	<.001

^a^Stage 3>stage 1>stage 2.

^b^Stage 3=stage 1>stage 2.

### Regression Analysis

To investigate whether there are any confounders, we also applied regression analysis. We built 4 regression models (*M_i_*, *i*=1-4). Each of them consecutively adds factor(s) to the model based on the previous one. The definition of *M_i_* is shown as follows, in which *y* denotes the dependent variable of the activity value, *b_j_* (*j*=0-5) are coefficients of the models, and *r* is residue. In addition, *group* is given to be a control variable and *stim* (stimulation, or the CPT) is given to be an independent variable. There are 2 potential confounders: *age* and *gender*. Table 6 shows that both the *group* and the *stim* are the only factors affecting the activity value (M_2_), and that there are no confounders in this study (M_3_ and M_4_).

*M_1_* : *y* = *b_0_* + *b_1_*(*group*) + *r*

*M_2_* : *y* = *b_0_* + *b_1_*(*group*) + *b_2_*(*stim*) + *r*

*M_3_* : *y* = *b_0_* + *b_1_*(*group*) + *b_2_*(*stim*) + *b_3_*(*age*) + *b_4_*(*gender*) + *r*

*M_4_* : *y* = *b_0_* + *b_1_*(*group*) + *b_2_*(*stim*) + *b_3_*(*age*) + *b_4_*(*gender*) + *b_5_*(age * gender) + *r*

**Table 6:** Regression models of the activity value respective to other variables.

Variable		Regression model										
		M_1_			M_2_			M_3_			M_4_	
		Coefficient	*P* value	Coefficient		*P* value	Coefficient		*P* value	Coefficient		*P* value
Control: group		–.0141	.052	–0.141		.006	–0.181		.001	–0.182		.001
Independent: stim^a^		N/A^b^	N/A	–0.707		<.001	–0.707		<.001	–0.707		<.001
**Confounder**												
	Age	N/A	N/A	N/A		N/A	–0.056		.273	–0.057		.265
	Gender	N/A	N/A	N/A		N/A	–0.079		.158	–0.080		.154
Interaction: Z_age_*Z_gender_		N/A	N/A	N/A		N/A	N/A		N/A	–.018		.722
*R* ^2^		0.020	N/A	0.520		N/A	0.528		N/A	0.528		N/A
∆*R*^2^		0.020	N/A	0.500		N/A	0.008		N/A	0.000		N/A
*F* (*df*)		3.814 (1,187)	.052	193.418 (1,186)		<.001	1.592 (2,184)		.206	0.127 (1,183)		.722

^a^*stim*: stimulation.

^b^N/A: not applicable.

## Discussion

This is a case-control study, but there are some limitations. Sample size and gender in the case and control groups are unmatched. To deal with the limitations, the study recruited an age-matched sample for both case and control groups. In addition, as recommended by Faresjö and Faresjö [23], we conducted multivariate regression analyses, using *gender* and *group* as covariates and testing for significance of coefficients. The analysis result shows that *group* is the only factor affecting the activity value, and that *gender* is not a confounder in the study. This study is the first to use both brain waves and activity monitors to help the diagnosis of ADHD. When participants with ADHD performed the CPT, the study found the new electrophysiological biomarker collected by the activity sensor to be especially useful. The result shows that the actigraphy is an objective tool for the monitoring of activity in patients with ADHD, supporting previous findings that use actigraphy to understand the features of children with ADHD [24-28]. However, Muñoz-Organero et al [27] used an exam time of 6 school hours, while this study took only 20 minutes, which provides a better performance.

Analytical results showed that correlation of brain waves and CPT indicators is significant only for hit RT ISI change and hit SE ISI change. However, CI, which is the most important indicator to diagnose ADHD, was not related to the brain waves. The problem probably came from the device. The participants reflected that it is inconvenient to wear, so much so that they felt uncomfortable during the test. In addition, even though the device protects against noise interference, some data were still found to have interference from poor electrode contact. The source of the noise was caused by the loosening of the brain wave device. With the interference, the attention and meditation values could not be continuously recorded. Even though the results support that stimulus on participants with ADHD is a mediated factor of response capability, they are similar to the findings on stimulus discriminability (two-choice reaction time task) [29,30], in which the stimulus improved ADHD participants' task performance and ability to differentiate optimal from nonoptimal choices [30].

In the end, previous studies [31-33] revealed that participants with ADHD (case group) have inhibition deﬁcits when compared with participants without ADHD (control group). However, in this study, ADHD participants presented lower activity in the stage with stimulus (the CPT) than in the stages without the stimulus. This implies that the extent of ADHD can be mediated by the stimulus. The study concludes that an activity recorder is a new electrophysiological biomarker that helps to diagnose ADHD. The use of an inappropriate recorder tends to get deviation results in activity values. Carefully selecting the recorder that can analyze the applicable scenarios and intensity of activities is vital. Furthermore, if the wearable device is worn on different parts of a person’s body, it will likely obtain different activity values. For future research, we suggest defining values for the threshold of activity for different ages, genders, and types of ADHD.

## Abbreviations

ADHD: attention-deficit/hyperactivity disorder
ANOVA: analysis of variance
CI: confidence index
CPT: continuous performance test
DSM-V: Diagnostic and Statistical Manual of Mental Disorders, Fifth Edition
EEG: electroencephalography
GDS: Gordon Diagnostic System
ISI: interstimulus interval change
RT: reaction time
